# A medium invasiveness multi-level patient’s specific template for pedicle screw placement in the scoliosis surgery

**DOI:** 10.1186/s12938-017-0421-0

**Published:** 2017-11-14

**Authors:** Farhad Azimifar, Kamran Hassani, Amir Hossein Saveh, Farhad Tabatabai Ghomsheh

**Affiliations:** 1 Department of Biomechanics, Science and Research Branch, Islamic Azad University, Tehran, Iran; 2 Functional Neurosurgery Research Center, Shohada Tajrish Neurosurgical Comprehensive Center of Excellence, Shahid Beheshti University of Medical Sciences, Tehran, Iran; 3 Pediatric Neurorehabilitation Research Center, Ergonomics Department, University of Social Welfare and Rehabilitation Sciences (USWR), Tehran, Iran

**Keywords:** Multi-level, Template, Spine, Surgery

## Abstract

**Background:**

Several methods including free-hand technique, fluoroscopic guidance, image-guided navigation, computer-assisted surgery system, robotic platform and patient’s specific templates are being used for pedicle screw placement. These methods have screw misplacements and are not always easy to be applied. Furthermore, it is necessary to expose completely a large portions of the spine in order to access fit entirely around the vertebrae.

**Methods:**

In this study, a multi-level patient’s specific template with medium invasiveness was proposed for pedicle screw placement in the scoliosis surgery. It helps to solve the problems related to the soft tissues removal. After a computer tomography (CT) scan of the spine, the templates were designed based on surgical considerations. Each template was manufactured using three-dimensional printing technology under a semi-flexible post processing. The templates were placed on vertebras at four points—at the base of the superior-inferior articular processes on both left–right sides. This helps to obtain less invasive and more accurate procedure as well as true-stable and easy placement in a unique position. The accuracy of screw positions was confirmed by CT scan after screw placement.

**Results:**

The result showed the correct alignment in pedicle screw placement. In addition, the template has been initially tested on a metal wire series Moulage (height 70 cm and material is PVC). The results demonstrated that it could be possible to implement it on a real patient.

**Conclusions:**

The proposed template significantly reduced screw misplacements, increased stability, and decreased the sliding & the intervention invasiveness.

## Background

The pedicle screw placement in scoliosis of the lumbar and thoracic spine has been extensively used in the surgical community [1]. The Pedicle screw fixation is used in spinal fusion surgery to fuse vertebrae together securely in a fixed position [2]. These devices provide stability and secure the spine after surgery and keep bone grafts in position during the spine recovery [3].

Correct placement of pedicle screws, in the lumbar and thoracic spine, needs to have a well 3D sensation and perception of the pedicle morphology for accurate identification of the ideal screw axis [4, 5]. It has been a problem, due to variations in anatomical shapes, dimension and orientation, which can cause the inefficiencies of treatment or severe injury to neurological structure [6, 7].

At the present time, the pedicle screw placements are applied by free-hand technique [8–13] or be performed under fluoroscopic guidance [14–19]. Other methods have also been conducted on a limited basis, such as image-guided navigation [20–25], computer-assisted surgery system [26–28], robotic platform [1, 29–32] and patient’s specific templates [33–45].

Free-hand procedure usage avoids any complexity but has error in rang 10–40% [46–48]. Image-guided navigation method reduces screw placement errors [49–52] but requires correct positioning and orientation of the drill to keep stability, which is not always easy to do. Furthermore, this technique is expensive and is used by a limited number of surgeons, due to the difficult challenges.

The robotic platform effectively reduces screw misplacement [53, 54] but it may not be a practical technique for small hospitals, which perform a limited number of spine fixation, due to the high cost and a long term learning process. According to studies conducted by Ludwig et al. [55], 18% of the pedicles of human cadaveric cervical spines, which were implanted with computer-assisted image-guided surgical system, had a critical violations.

Another different approach, which is cheaper with less complexity, could be the use of patient’s specific templates [56]. These templates are very similar to dental implants surgical-guides which help to guide the drill in right direction. Nevertheless, in most designs, it is necessary to remove a huge region of the soft tissues and expose completely a large portions of the spine in order to access fit entirely around the vertebrae [37, 41].

A comprehensive literature review has been performed on various aspects of Patient’s specific templates. It is evident from the literature review that there is approximately no report on the design of templates by considering three factors including stability, medium invasiveness and easy verification, simultaneously. The outcome of our study would be extremely useful as the technology charts for designing medium invasiveness patient’s specific templates which are false-stable and not easily verifiable for pedicle screw placement in the spine.

## Methodology

**Fig. 1:**
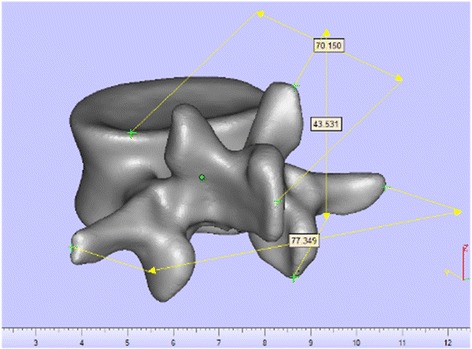
Evaluation file quality, detection bad edges, flipped triangles and multiple shells and making single point to point measurements performed by MiniMagics 3.0 software

**Fig. 2:**
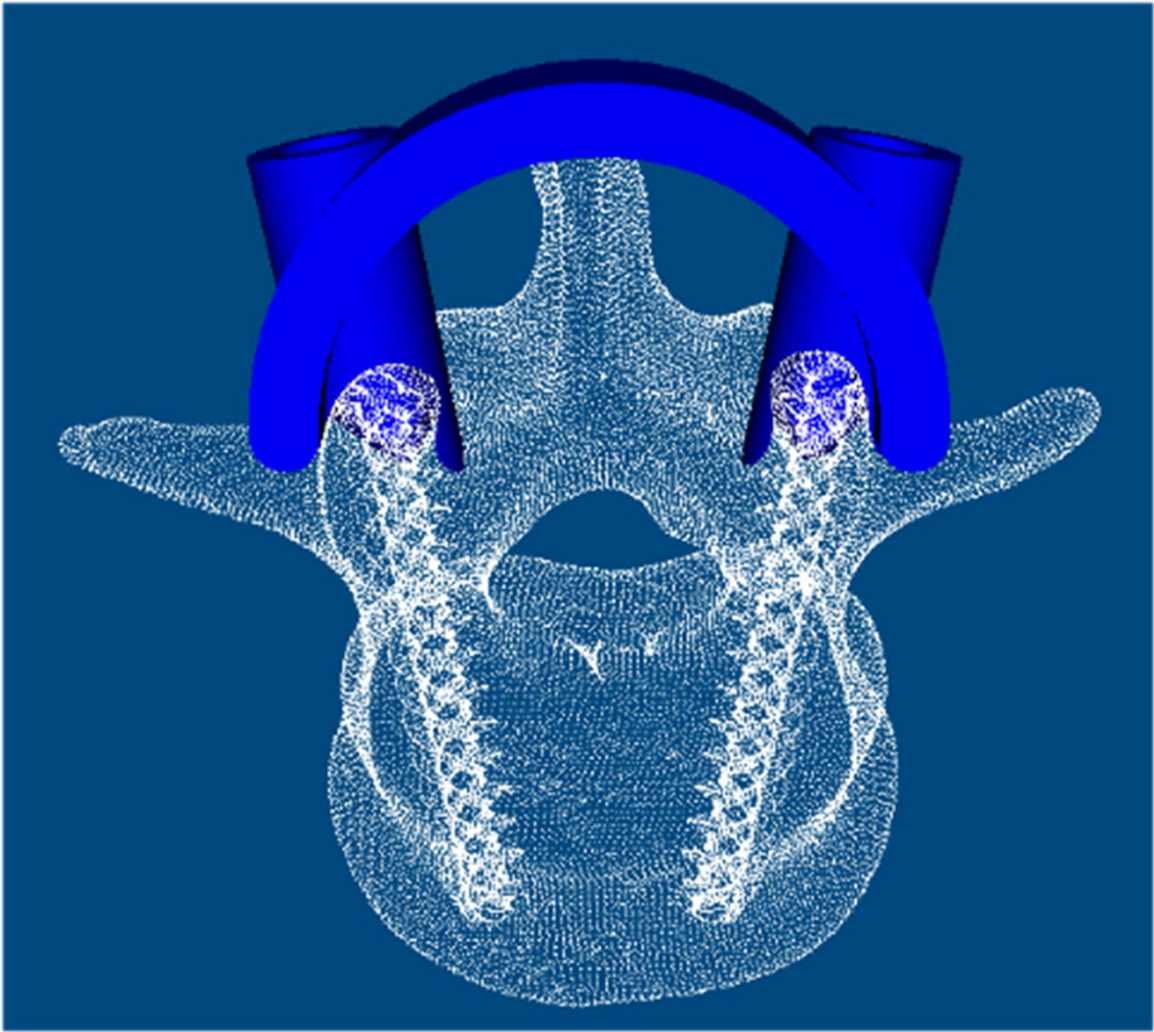
3D modeling of the templates and modification of the anatomical data performed by 3-matic Medical 9.0 software

**Fig. 3:**
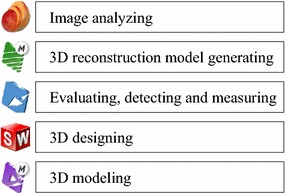
Templates design process

A multi-slice 64 bit 3D CT scan (Somatom Definition, Siemens, Germany) was performed on 12 scoliosis patients (8 females, 4 males, age 6–20 years) with 0.625 mm slice thickness and 0.35 mm in-plane resolution. The subjects have been complied by the World Medical Association Declaration of Helsinki regarding ethical conduct of research involving human subjects. The images were stored in DCM format and analyzed in Mango (Multi-Image Analysis and Navigation GUI). Mimics Medical 17.0 software (Materialise, Belgium) was used to generate a 3D reconstruction model. The 3D vertebral model was exported in STL format for MiniMagics 3.0 software (Materialise, Belgium) in order to evaluate file quality, detect bad edges, flipped triangles and multiple shells, and make single point to point measurements (Fig. 1). The arc-shape part and the hollow cylinders of templates were designed in a 3D CAD Design Software (Dassault Systèmes, SOLIDWORKS Corp). As can be seen in Fig. 2, 3D modeling of templates and modification of the anatomical data were performed by 3-matic Medical 9.0 software (Materialise, Belgium). The process of templates design is shown in Fig. 3.

**Fig. 4:**
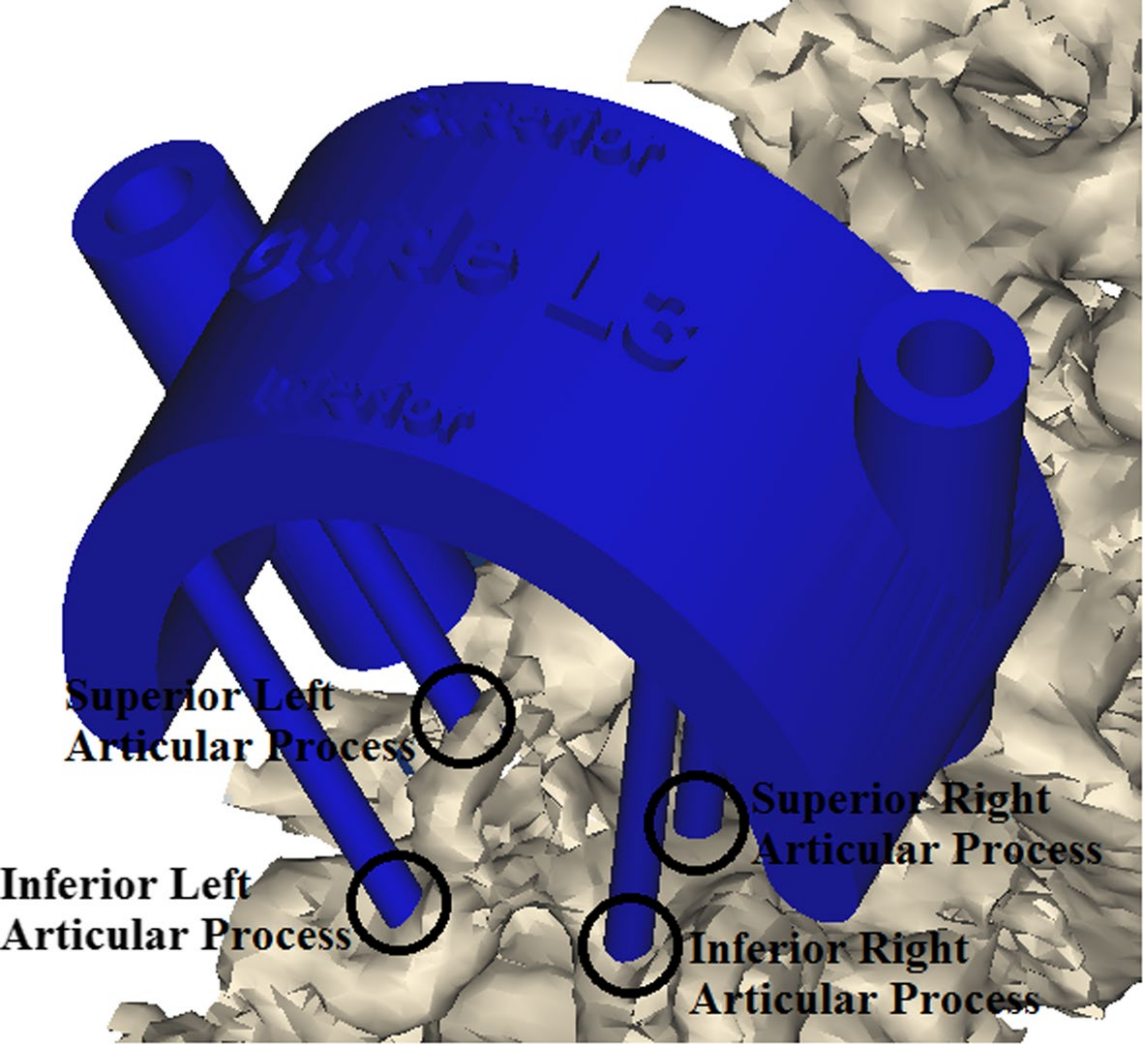
Template perspective view. There are 4 points at the base of the superior-inferior articular processes on both left–right sides that help to easy template alignment and simplify template correct positioning

The important factor in template design is a stable fit on the bony structures. To obtain the template stability and avoid sliding, it is necessary to expose completely a large portions of the spine in order to access fit entirely around the vertebrae. It leads to increase intervention invasiveness. The presented design aims to establish a balance between invasiveness and template stability noting to 4 points—at the base of the superior-inferior articular processes on both left–right sides as the supporting points (Fig. 4). Because of two main reasons, the spinous and transverse processes should not be used as the supporting points:

1. The exact anatomical size of supra-spinous ligament, that covered the bone, is not clear.
2. The use of transverse processes requires a larger bone exposure.

Failure to use these points causes instability. The result of this study can fix this defect using designed multi-level template. Therefore the remaining required supporting points have been considered on another spinal level at the base of the articular processes.

**Fig. 5:**
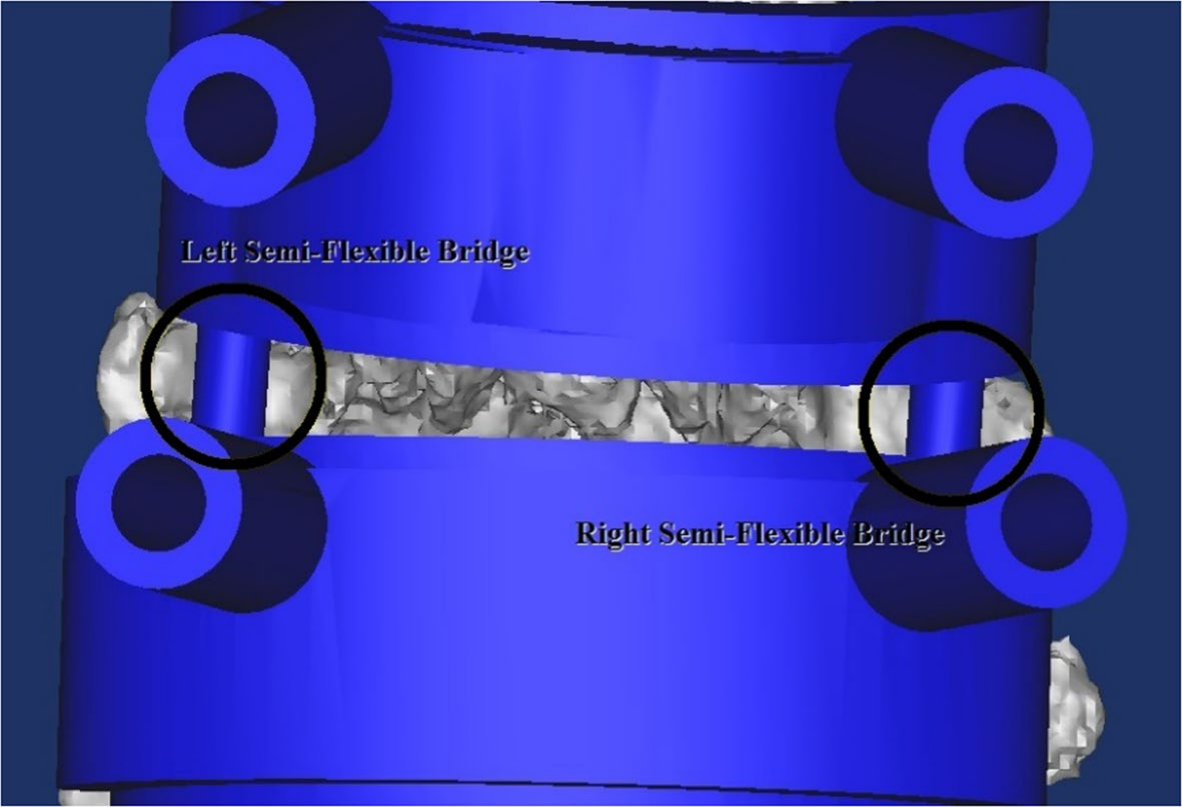
Semi-flexible bridges between different levels in multi-level templates. The semi-flexible bridges were significantly reduced error rate, due to the changes in the relationship between each vertebral bodies

The use of multi-level designs is associated to a low accuracy level, due to the changes between each vertebral bodies of the CT acquisition and the surgical table [42, 57, 58]. Since, the patient’s position during operating is prone, then the CT scan protocol has also been performed on prone position in order to simulate equal facet joint relations during the operating. This solution provide a good accuracy for multi-level templates. Furthermore, the connections (bridges) between different levels of designed template have been processed under a semi-flexible post processing (Fig. 5).

The design should guide the surgeon how to use the template. The application of the current available templates can be difficult, due to a lack of easy verification of correct or incorrect positioning. In this study, each template has been manufactured using 3D printing technology and specifically fused deposition modelling (FDM), under a transparent post processing, in order to achieve appropriate identification of correct positioning (Table 1).

**Table 1:** Literature review (tabular form)

No.	Sr. investigator	Design technique	Edge shape	Level	Location of the supporting points	Invasiveness	Stability	Verification
1	Tominc et al. [33]	Low contact	Fit and lock on the lamina	1st to 7th thoracic vertebrae	On the lamina at the base of the superior articular process on both sides and at the tip of the spinous process	Medium	Good	Moderate
2	Ferrari et al. [34]	Low contact	Complementary likeness of the bone surface	Thoracic and lumbar vertebrae	On the lamina, articular and spinous processes	Medium	Good	Moderate
3	Ma et al. [35]	Full contact	Inverse of the vertebral posterior surface	1st to 12th thoracic vertebrae	On the lamina, superior articular, spinous and transverse processes	High	Perfect	Easy
4	Lu et al. [36]	Full contact	Inverse of the spinous, lamina, and transverse processes	2nd to 12th thoracic vertebrae	On the spinous process, lamina, and transverse processes	High	Perfect	Easy
5	Lu et al. [37]	Full contact	Inverse of C2 spinous process and lamina	2nd cervical vertebrae	On the spinous process, lamina, and lateral masses of C2	High	Perfect	Easy
6	Lu et al. [38]	Full contact	Inverse of the vertebral posterior surface	2nd to 7th cervical vertebrae	On the spinous process and lamina	High	Perfect	Easy
7	Lu et al. [39]	Full contact	Inverse of the vertebral posterior surface	12th thoracic to 5th lumbar vertebrae	On the posterior surface of the lumbar vertebra	High	Perfect	Easy
8	Ryken et al. [40]	Full contact	Inverse of the vertebral surface	5th to 6th cervical vertebrae	On the lamina, spinous and transverse processes	High	Perfect	Easy
9	Ryken et al. [41]	Full contact	Inverse of the vertebral surface	2nd cervical to 1st thoracic vertebrae	On the spinous process and lamina	High	Perfect	Easy
10	Berry et al. [42]	Low contact	V-shape	Cervical, thoracic and lumbar vertebrae	On the posterior surface of the lamina plus a posterior support to fit the spinous process	Medium	Good	Difficult
11	Goffin et al. [43]	Full contact	Inverse of the spinous process and lamina	1st and 2nd cervical vertebrae	On the spinous process and the left and right side of the lamina	High	Perfect	Easy
12	Porada et al. [44]	Low contact	V-shape	1st to 4th lumbar vertebrae	On the surfaces of a vertebra’s transverse and spinous processes	Medium	Good	Difficult
13	Van Brussel et al. [45]	Low contact	knife-edge	2nd to 4th lumbar vertebrae	A large contact area on the top of the spinous process and a small knife-edge area on the two transverse processes	Medium	Good	Difficult

FDM is a popular RP technology, widely used in industries, to build complex geometrical functional parts in short time [59, 60]. FDM process includes applications ranging from prototype to functional parts. Creation of CAD model, conversion of CAD model into STL format, slicing of STL format into thin layers, construction of part in layer-by-layer fashion and cleaning and finishing are the five simple steps used in FDM process to manufacture a part. In Table 2, the 3D model and 3D printer parameters, related to the production, are given:

**Table 2:** 3D model and 3D printer parameters related to the production

3D model parameters		3D printer parameters	
Profiles	Valve	Profiles	Valve
Layer height	0.25	Nozzle size	0.4
Wall thickness	0.8	Print speed	30
Retraction enable	True	Print temperature	215
Solid layer thickness	1	Print bed temperature	45
Fill density	50	Support	Everywhere
Layer thickness	0.06	Platform adhesion	None
Fill angle	60	Support dual extrusion	Second extruder

In order to consider the strength of the designed template during surgery, the optimal conditions in fabrication parameters of FDM machine have been obtained to improve the strength of shafts. Two variables have remarkable impact on the fabricated product’s strength in the FDM machine. These two variables are Layer thickness and Fill angle [61].

Therefore, before fabricating drill guide templates, different samples regarding various conditions, mentioned in [61], were fabricated via FDM machine. Consequently, the optimal parameters which can achieve maximum strength were specified. Note that the values of 0.06 and 60 have been considered for layer thickness and fill angle respectively.

Regarding the considered values, the maximum (UTS) of each shaft is 38.67 MPa and the diameter of each shaft has been considered 5 mm. Therefore, it is obvious that every shaft can tolerate 75.8 N. Note that, 4 shafts were used for every drill guide template.

Moreover, based on information associated with force applied by surgeon on the drill guide template during surgery, the applied force would be variable between 30 and 50 N. Therefore, it could be concluded that the designed shafts would not be broken during surgery.

In addition, the orientation of shafts has been designed to guarantee drill guide template during surgery. Finally, the experimental condition demonstrated that the drill guide template is stable during surgery and can tolerate surgeon force properly.

## Results and discussion

As mentioned in the introduction section, using free-hand technique for pedicle screw placement is followed by high risk of screws miss positioning [46–48]. Image-guided navigation method is expensive and not always easy task to be performed [49–52]. The robotic platform is not often used because of its high cost and difficulty in learning [53, 54].

**Fig. 6:**
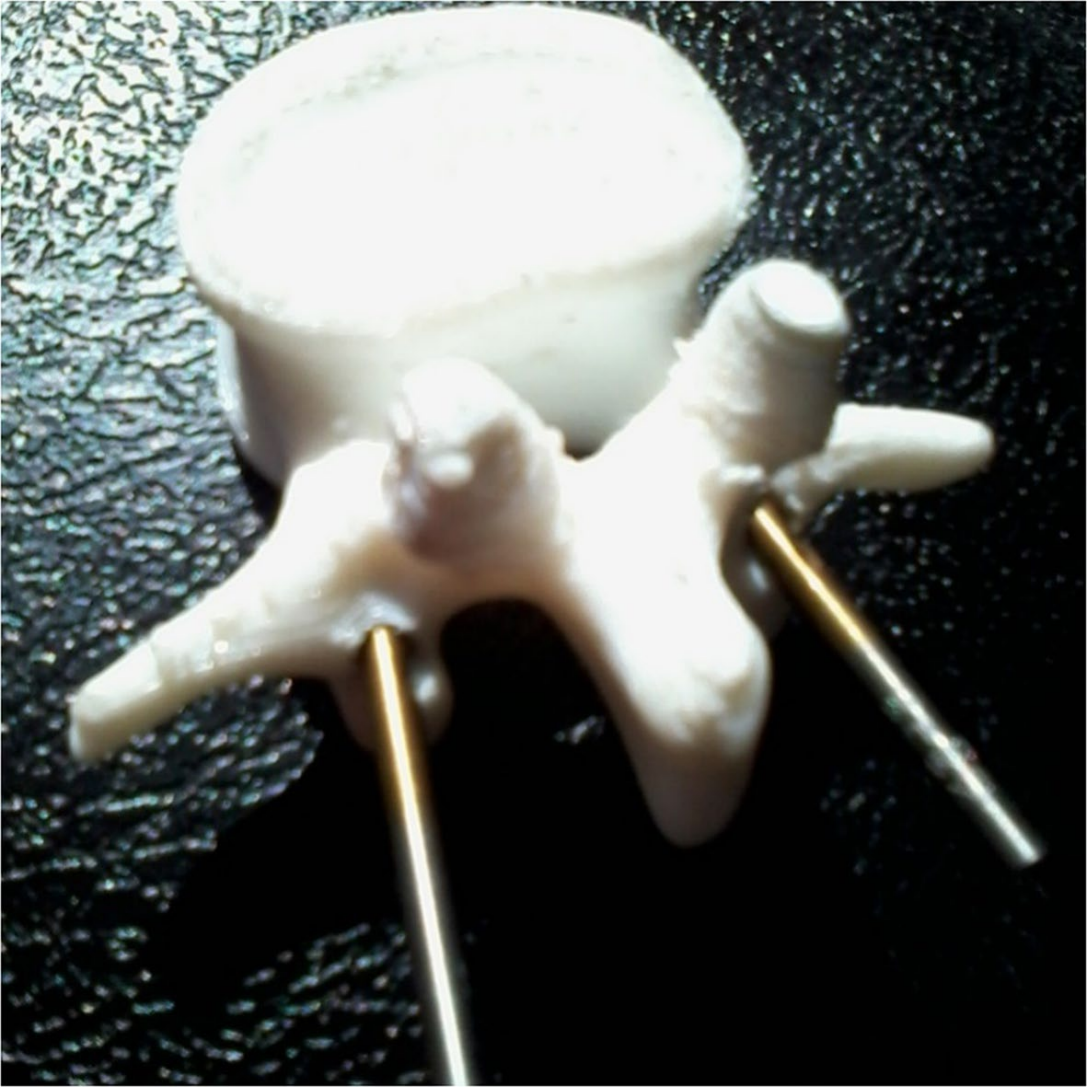
The template was tested on an ex vivo test (3D printed model). The result showed the correct alignment in pedicle screw placement

**Fig. 7:**
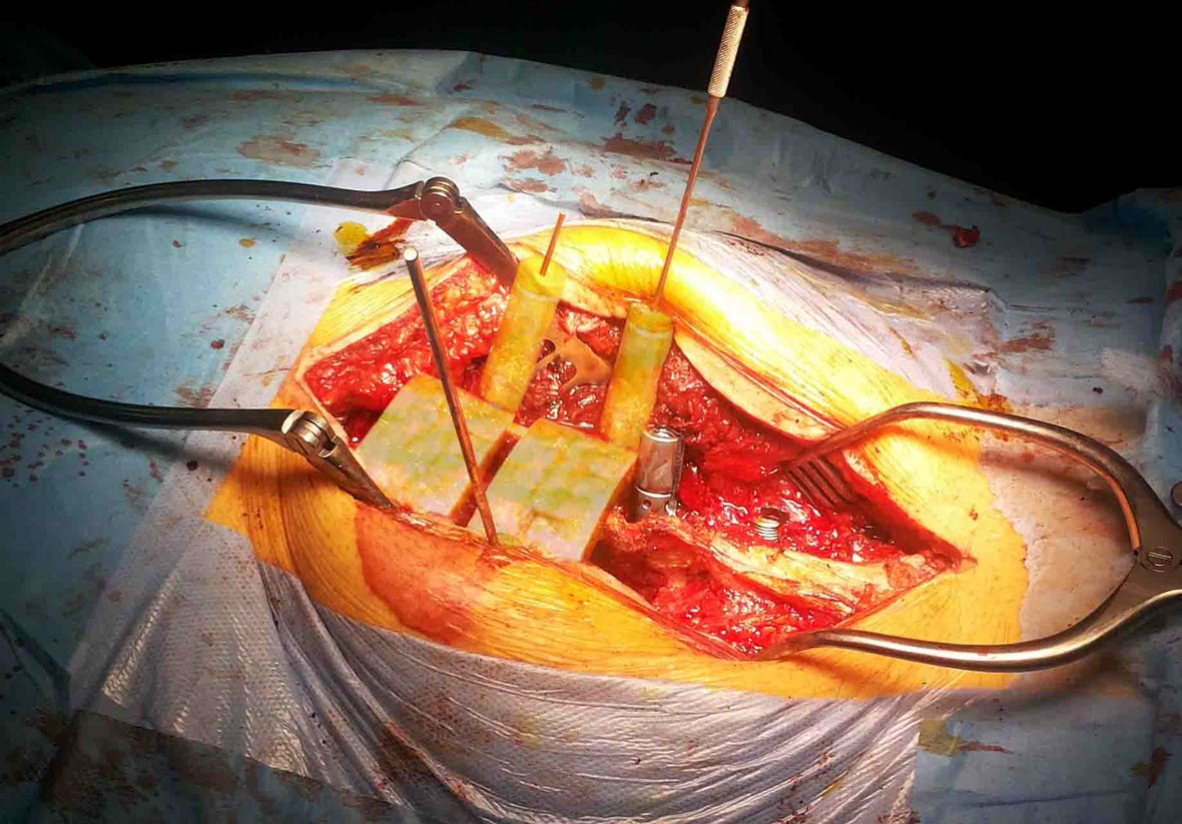
12-year-old male with a 62° pre-operative curve [an in vivo trial]. Lumbar pedicle screw was inserted using the template. The template fits the posterior part of the lumbar perfectly in the operation

Based on these studies, another different method, which is cheaper and easier, could be the use of patient’s specific templates [56]. Application of this approach will be recommended if balancing is noted between invasiveness and template stability. It is evident from the literature review that there is approximately no report on the design of templates by considering three factors including stability, medium invasiveness and easy verification, simultaneously. In this study, the balance between these factors has been achieved noting to 4 points—at the base of the superior-inferior articular processes on both left–right sides as the supporting points. As shown in Fig. 6, the template was tested on a 3D printed model. The result showed the correct alignment in pedicle screw placement. In addition, the template has been initially tested on a metal wire series Moulage (height 70 cm and material is PVC). The results demonstrated that it could be possible to implement it on a real patient. A total of 12 multi-level templates were successfully applied in vivo (Fig. 7). Templates were perfectly fixed on the articular processes surface by small and thin-fitting areas without increasing intervention invasiveness.

**Fig. 8:**
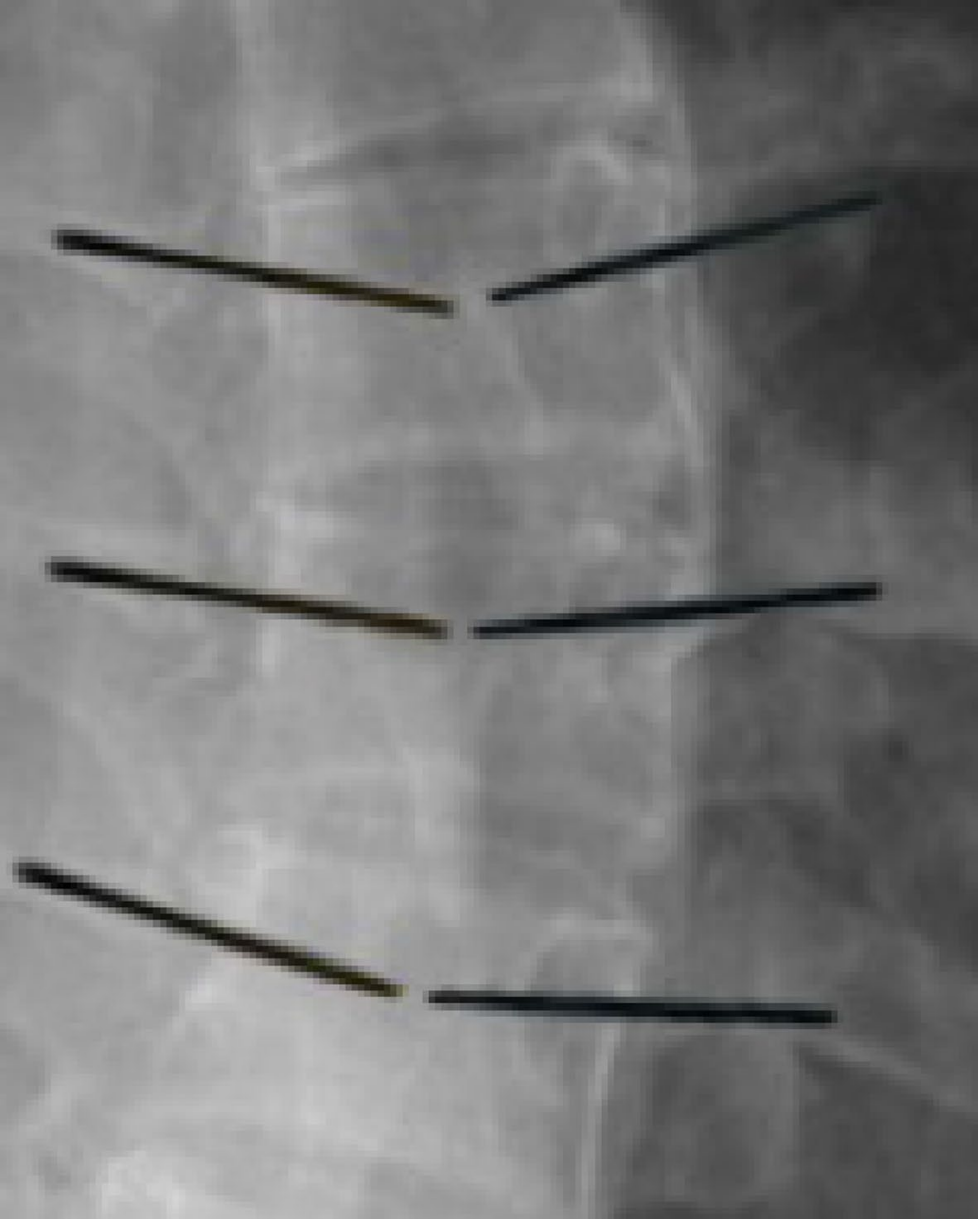
The template was successful applied in vivo trials. The postoperative CT evaluation (performed after each trial session) showed an error less than 1 mm in 94% of the cases and between 1–2 mm in 6% of the cases

As it mentioned, the spinous and transverse processes should not be used as the supporting points, due to the problems related to the soft tissues removal. However, these listed points have been mistakenly used in most studies. The supporting points on another spinal level, at the designed multi-level template, has fixed this problem. The use of designed template, in 12 scoliosis surgeries, helped to reduce the rate of screws misplacement, due to template stability. The post-operative CT evaluation showed that 103 screws (94%) were implanted accurately (Fig. 8).

Note that, 7 out of 103 screws, were implanted correctly but with less accurately (between 1 and 2 mm). This error can be ignored using free-hand procedure that has error in range 10–40% [46–48]. Therefore, it could be claimed that the obtained results, compared to previous studies, are acceptable. The errors are caused due to variations in anatomical shapes, dimension, and orientation in scoliosis cases. In addition, manufacturing process by 3D printing has dimensional errors because of the shrinkage during fabrication. Moreover, complex geometry, in the spine due to scoliosis, could lead to make error during process.

The results of surgery, testing for 110 screws, are given in Tables 3 and 4, respectively.

**Table 3:** Results of surgery testing for 110 screws

	Number of screws	%
Trajectory error < 1 mm	103	93.63
Trajectory error between 1 and 1.5 mm	4	3.63
Trajectory error between 1.5 and 2 mm	3	2.72

**Table 4:** Distribution by vertebra of the screws that violated the pedicular cortex

Vertebra	Total number of screws	Misplaced screws (n)	Misplaced screws (%)^a^
T1	0	0	0
T2	1	0	0
T3	1	0	0
T4	3	1	0.9
T5	3	0	0
T6	3	0	0
T7	4	1	0.9
T8	2	0	0
T9	5	0	0
T10	7	1	0.9
T11	10	0	0
T12	12	2	1.81
L1	11	0	0
L2	12	0	0
L3	12	2	1.81
L4	10	0	0
L5	9	0	0
S1	5	0	0

^a^This value actually represents the misplaced screws percentage of the total 110 screws

**Fig. 9:**
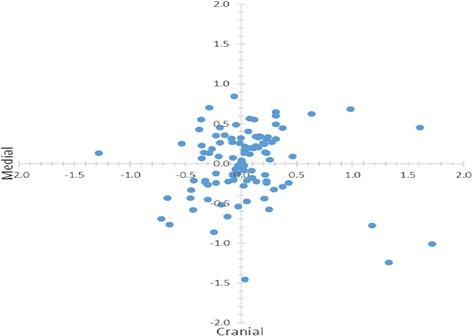
Displacement of screws in cranial and medial plane according to the predefined screw placement in the center of the pedicle

The statistical analysis were performed using Minitab 17 statistical software. Descriptive statistic were used to analyze the obtained data. The inaccuracy of screws were presented in percent, as mean value and standard deviation (SD). The learning curve for the application of multi-level templates was estimated using Pearson correlation. The analysis showed that the average final position of screws was less lateral and caudal than predicted. The deviation measurements were revealed that the screws were directed less medially and caudally in comparison to the predefined direction (Fig. 9).

**Fig. 10:**
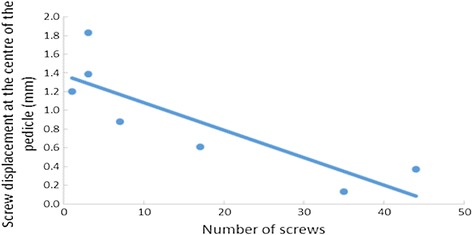
Correlation of screw displacement rate according to the number of screw placement

Pearson correlation estimated that the smaller group of screws has a larger values of screw displacement at the center of the pedicle (Fig. 10).

All the multilevel templates, manufactured until now, have been associated to a high error rate due to the changes of vertebral bodies from the CT acquisition to the surgical table [42, 57, 58]. In this study, to provide a good accuracy for multi-level templates, the CT scan of spine was performed on patients lying in prone position to simulate equal facet joint relations during the operating. The bridges, between different levels of designed template, have also been processed under a semi-flexible post processing in order to achieve fit ideally on two or more vertebrae in vivo. This technique were significantly reduced the problems related to the changes occurred in each vertebral body.

To achieve easy verification of correct positioning, templates has been processed under a transparent post processing. During the in vivo trials, the surgeon found that template alignment was easy, due to the transparency of templates. We have found two previous research which have relatively similar results to us [62, 63]. Ferrari et al. [62] designed patient-specific templates for pedicle spine screws placement but their method has some limitations: the method is suitable only for single guide but ours used multi-level guides which have much superiority to single ones regarding less invasiveness, less surgery time, and less bone exposures. We also see that their proposed method suggests to fit the guide on all main areas of vertebra including spinous processes, lamina, and articular process. This leads to eliminate of considerable soft tissues. Our method has used only anterior and posterior articular process symmetrically which prevents to eliminate much soft tissues. These symmetrical areas could prevent the sliding of the guides and neutralize its freedom degrees without elimination of lamina and spinous soft tissues. Therefore, we think that our method is less invasive than Ferrari’s method. The most important point is that Ferrari’s proposal is not suitable for multi-level guides and scoliosis. The reason is that they have used drilled bushings, as supports on the vertebras, to keep stability. In our system, there is no any physical contact between bushing and vertebra surface. Therefore, it is possible to disregard the damaged vertebras, or the vertebras with abnormal geometries, as supporting areas (reference).

Putzier et al. tool for pedicle screw placement in patients with severe scoliosis [63] was also compare to our study and there are some issues as below:

1. They have used spinous process, both lamina, and transverse processes as contact points but these points are not suggested due to following reasons:
  - The anatomical data of spinous process can not be specified due to supra-spinous. Therefore, we think that spinous process can not be used as contact point.
  - It is correct that usage of transverse processes and lamina increases the stability but a considerable amount of soft tissue shall be eliminated. Therefore, these could not be suggested as contact points.
    We have used inferior articular process as contact point which avoids the above mentioned problems.
2. One of the weak points of that work is that the size of the guide is not suitable for multi-level in vivo surgeries. They have mentioned in result section that the guide has hit to L4 screw head due to its big size and they have used fluoroscopy method instead to fix the screws. In general, the big guides normally hit to the screws and other equipment and verification of the process is not easy. Furthermore, the method is not user friendly for surgeons. The big guide leads to elimination of much soft tissues as well.
3. The accuracy of their method for fixing the screws was 84% but our method’s accuracy is 96%.
4. The most important point in design of the guides, which are used in in vivo surgeries, is the geometry and size of the guide. In in vivo surgeries, the expose of soft tissue is an important factor concerning the time of surgery and related infection. Therefore, big size guides are only suitable for single level method not multi-level one. The multi-level guides could lead to minimum tissue expose.

## Conclusions

The presented study demonstrated a medium invasiveness multi-level patient’s specific template for pedicle screw placement, which is easy to apply in the scoliosis surgery. The following conclusions are drawn from the present investigation:

1. With respect to the design principles, under the surgical considerations and noting to small and thin-fitting areas, it is feasible to decrease intervention invasiveness.
2. Using additional supporting points on another spinal level, at the multi-level templates, decreases the possibility to have a false-stable template positions.
3. Semi-flexible bridges, between different levels in multi-level templates, significantly reduce error rate due to the changes in each vertebral bodies.
4. Transparent multi-level patient’s specific template is easy to apply and give hint to the surgeon how to use the template correctly.

The outcome of this study would be extremely useful as the technology charts for designing medium invasiveness patient’s specific templates which are false-stable and not easily verifiable for pedicle screw placement in the scoliosis surgery.
